# Regulation of soluble guanylate cyclase by matricellular thrombospondins: implications for blood flow

**DOI:** 10.3389/fphys.2014.00134

**Published:** 2014-04-04

**Authors:** Natasha M. Rogers, Franziska Seeger, Elsa D. Garcin, David D. Roberts, Jeffrey S. Isenberg

**Affiliations:** ^1^Department of Medicine, Vascular Medicine Institute, University of Pittsburgh School of MedicinePittsburgh, PA, USA; ^2^Department of Chemistry and Biochemistry, University of Maryland Baltimore CountyBaltimore, MD, USA; ^3^Laboratory of Pathology, Center for Cancer Research, National Cancer Institute, NIHBethesda, MD, USA; ^4^Department of Medicine, Division of Pulmonary, Allergy and Critical Care Medicine, University of Pittsburgh School of MedicinePittsburgh, PA, USA

**Keywords:** soluble guanylate cyclase, cyclic guanosine monophosphate, thrombospondin-1, CD47, nitric oxide, cardiovascular disease, ROS

## Abstract

Nitric oxide (NO) maintains cardiovascular health by activating soluble guanylate cyclase (sGC) to increase cellular cGMP levels. Cardiovascular disease is characterized by decreased NO-sGC-cGMP signaling. Pharmacological activators and stimulators of sGC are being actively pursued as therapies for acute heart failure and pulmonary hypertension. Here we review molecular mechanisms that modulate sGC activity while emphasizing a novel biochemical pathway in which binding of the matricellular protein thrombospondin-1 (TSP1) to the cell surface receptor CD47 causes inhibition of sGC. We discuss the therapeutic implications of this pathway for blood flow, tissue perfusion, and cell survival under physiologic and disease conditions.

## Introduction

Cardiovascular disease is the number one cause of death worldwide and remains a burdensome health problem. Understanding the underlying molecular mechanisms of cardiovascular disease will provide valuable information for the design of novel drugs for pharmacological therapies.

As a central player of the canonical NO-sGC-cGMP pathway, sGC controls important physiological functions such as smooth muscle relaxation, platelet aggregation, and hemostasis (Isenberg et al., 2009b). Hence, sGC activity is a primary means of controlling acute and chronic blood flow. The gaseous molecule NO directly activates sGC and increases the production of the signaling molecule 3',5'-cyclic GMP (cGMP) from GTP several hundred fold (Arnold et al., 1977). Second messenger cGMP, in turn, targets the contractile apparatus of arterial smooth muscle cells and adhesive pathways of platelets and inflammatory cells to promote vasodilation and disadhesion/disaggregation (Murad et al., 1985; Munzel et al., 2003). Decreased NO bioavailability and sGC oxidation lead to inefficient sGC activation and lower cGMP output, which are associated with and directly contribute to cardiovascular diseases including ischemic heart disease, decompensated heart failure, ischemia reperfusion injury, visceral organ transplant failure, stroke, pulmonary and systemic hypertension, and atherosclerosis (Murad et al., 1978; Lucas et al., 2000; Loscalzo, 2001; Voetsch et al., 2004; Mitrovic et al., 2011).

Here, we first review molecular factors that modulate sGC activity under healthy and diseased conditions. The sGC enzyme is tightly regulated by its oligomeric and conformational state, endogenous and pharmacological ligands, post-translational modification, the cellular redox environment, sub-cellular localization, and protein-protein interactions. We then highlight the role of matricellular protein signaling, in particular TSP1, in inhibiting sGC activity and its potentially important implications for angiogenesis and blood flow.

## The NO-sGC-cGMP pathway in the cardiovascular system

Physiologic (low dose) NO is formed via two primary pathways. NO is generated when endothelial nitric oxide synthase (eNOS) is activated by Ca^2+^/calmodulin binding in response to external stimuli including laminar blood flow and hormonal cues, such as vascular endothelial growth factor (VEGF) (Zhao et al., 1999). Another source of NO arises from the reduction of dietary nitrates to nitrite and eventually NO. This latter source of NO is eNOS-independent and is likely relevant in conditions of hypoxia (reviewed in Lundberg et al., 2008; Weitzberg and Lundberg, 2013). The half-life of NO under physiological conditions is roughly 0.1 s (Kelm and Schrader, 1990) and its affinity for sGC is in the picomolar to low nanomolar range (Stone and Marletta, 1996; Tsai et al., 2012a), indicating that sGC is highly responsive to low NO concentrations. NO binding to sGC increases enzyme activity several hundred fold by inducing a transition from a basal to an activated state (Moncada and Higgs, 1993). Downstream of sGC, cGMP interacts with two different classes of effector molecules to achieve vascular smooth muscle relaxation: cGMP-dependent kinase (cGKs) and phosphodiesterases (PDEs) (Lincoln and Cornwell, 1993; Wu et al., 2000). The final cellular concentration of cGMP depends on the rate of production by sGC and the rate of hydrolysis by cGMP (PDEs).

## sGC is a modular heterodimeric enzyme

Cytoplasmic sGC exists as a heterodimer of an α and a β subunit. Heterodimerization is crucial for catalytic activity of sGC since both subunits contribute residues to the active site that is formed at the interface of the catalytic domain (Buechler et al., 1991). However, homodimers exist *in vitro* and cell lysates (Zabel et al., 1999) and certain functions have been attributed to a single subunit *in vivo* (Gao et al., 2013). It has been further proposed that homo- and heterodimerization may regulate activity *in vivo* and that inactive homodimer pools may regulate the formation of active and activatable heterodimers within the cell (Zabel et al., 1999; Zhou et al., 2008).

Mammals have two different isoforms of each sGC subunit. The α_1_β_1_ sGC heterodimer is the best characterized and is the predominant form in the cardiovascular system (Gupta et al., 1997; Mergia et al., 2003). The regulatory N-terminal domain of the β subunit harbors a heme prosthetic group that is the primary NO binding site of the enzyme (Gerzer et al., 1981). The high-resolution three dimensional structure of sGC is unknown but crystal structures of individual domains or domain homologs have been determined for the HNOX domain (Nioche et al., 2004; Pellicena et al., 2004; Ma et al., 2007; Olea et al., 2008; Erbil et al., 2009; Martin et al., 2010; Olea et al., 2010; Weinert et al., 2010, 2011; Winter et al., 2011), the HNOXA domain (Ma et al., 2008; Purohit et al., 2013), the CC domain (Ma et al., 2010), and the GC domain (Rauch et al., 2008; Winger et al., 2008; Allerston et al., 2013; Seeger et al., 2014). Recent studies suggest how these domains assemble in space to form the full-length enzyme. Winger et al., first suggested that βHNOX directly binds to and inhibits the cyclase domains (Winger and Marletta, 2005). Later studies supported this hypothesis, and further showed close proximity of the βHNOX and cyclase domains (Haase et al., 2010; Underbakke et al., 2013; Busker et al., 2014). Recent studies also demonstrated that αHNOX and αHNOXA maintain the βHNOX in an inhibited state that is released upon NO/activator binding thus leading to cyclase activation (Fritz et al., 2013; Purohit et al., 2014). Complementing this model of auto-inhibition, a comprehensive regulation mechanism was recently proposed whereby the activity of sGC is fine-tuned by distinct domain interactions that either inhibit or promote an optimal conformation of the active center (Seeger et al., 2014). Low-resolution electron microscopy (EM) data on rat sGC confirm most previous observations regarding the domain arrangement of sGC (Campbell et al., 2014). The EM envelope shows two distinct lobes comprising the HNOX and HNOXA domains at the N-terminus and the GC domains at the C-terminus. These two lobes are connected by a parallel CC domain linker. The reconstruction suggests that the full-length enzyme is highly flexible around the HNOXA-CC and CC-GC domain borders and explore a wide range of conformational space. Substrate and/or NO binding to the enzyme do not seem to stabilize specific conformations or restrict the movement observed in the apo enzyme. The lack of observation of distinct conformations that may correspond to the basal and activated state of the enzyme leads to the conclusion that domain-domain interactions as well as small intra-molecular changes account for the transition between the two activity states of sGC (Campbell et al., 2014; Seeger et al., 2014). Despite these significant advances, the exact mechanism by which sGC propagates the NO activation signal from the regulatory N-terminus to the catalytic C-terminus of the protein remains elusive (reviewed in Derbyshire and Marletta, 2012; Fritz et al., 2013; Underbakke et al., 2013).

## sGC activation by NO

When NO binds to the β subunit heme of sGC, a complex is formed in which both NO and β-His^105^ axially ligate the Fe^2+^ atom (Stone et al., 1995; Stone and Marletta, 1996; Zhao et al., 1999; Goodrich et al., 2010). This NO binding event leads to elongation and possibly breakage of the Fe-His^105^ bond and formation of the NO-bound sGC species (Dierks et al., 1997). Subsequent structural rearrangements in the enzyme lead to a 100–200 fold increase in enzyme activity (Wedel et al., 1994; Russwurm and Koesling, 2004; Cary et al., 2005; Pal and Kitagawa, 2010).

The first NO binding event is instantaneous (Stone and Marletta, 1996). Subsequent decay of the NO-sGC-His^105^ complex can form two catalytically distinct species: a high- and a low-activity NO-sGC (Russwurm and Koesling, 2004; Cary et al., 2005; Derbyshire et al., 2008). In conditions of excess NO or stoichiometric NO in the presence of substrate or product, the fully active NO-sGC species is formed (Russwurm and Koesling, 2004; Cary et al., 2005; Tsai et al., 2012a). In conditions of stoichiometric amounts of NO in the absence of substrate or products, a highly active NO-sGC species is formed that then rapidly degrades into a low-activity NO-sGC species (characterized by cGMP formation levels only a few fold above the basal level) (Tsai et al., 2011).

## Potential mechanisms of sGC desensitization

Interestingly, once exposed to NO, sGC decreases its cGMP output during subsequent NO exposure—a process termed desensitization (Mulsch et al., 1988; Schroder et al., 1988). Three potential mechanisms may be responsible for sGC desensitization: (i) heme oxidation (Schrammel et al., 1996; Zhao et al., 2000), (ii) heme nitrosylation by a second NO molecule (Tsai et al., 2011), or (iii) S-nitrosation of Cys residues in close proximity to the heme pocket (Sayed et al., 2007; Fernhoff et al., 2009; Mayer et al., 2009; Baskaran et al., 2011). The deactivation of sGC—the transition from the activated to the basal/sensitized state—is not as well understood as the process of sGC activation. Whether the dissociation of NO from the enzyme directly leads to deactivation remains controversial: *in vitro*, deactivation occurs within minutes after NO dissociation (Kharitonov et al., 1997; Brandish et al., 1998); in contrast, *in vivo*, NO dissociation leads to enzyme deactivation within seconds (Bellamy et al., 2000). sGC stimulators slow down sGC deactivation (Russwurm et al., 2002). Other sGC activity regulators such as nucleotides do not appear to influence the deactivation mechanism (Margulis and Sitaramayya, 2000; Russwurm et al., 2002).

## Nucleotides, small molecules, and calcium inhibit sGC activity and activation

**Figure 1:**
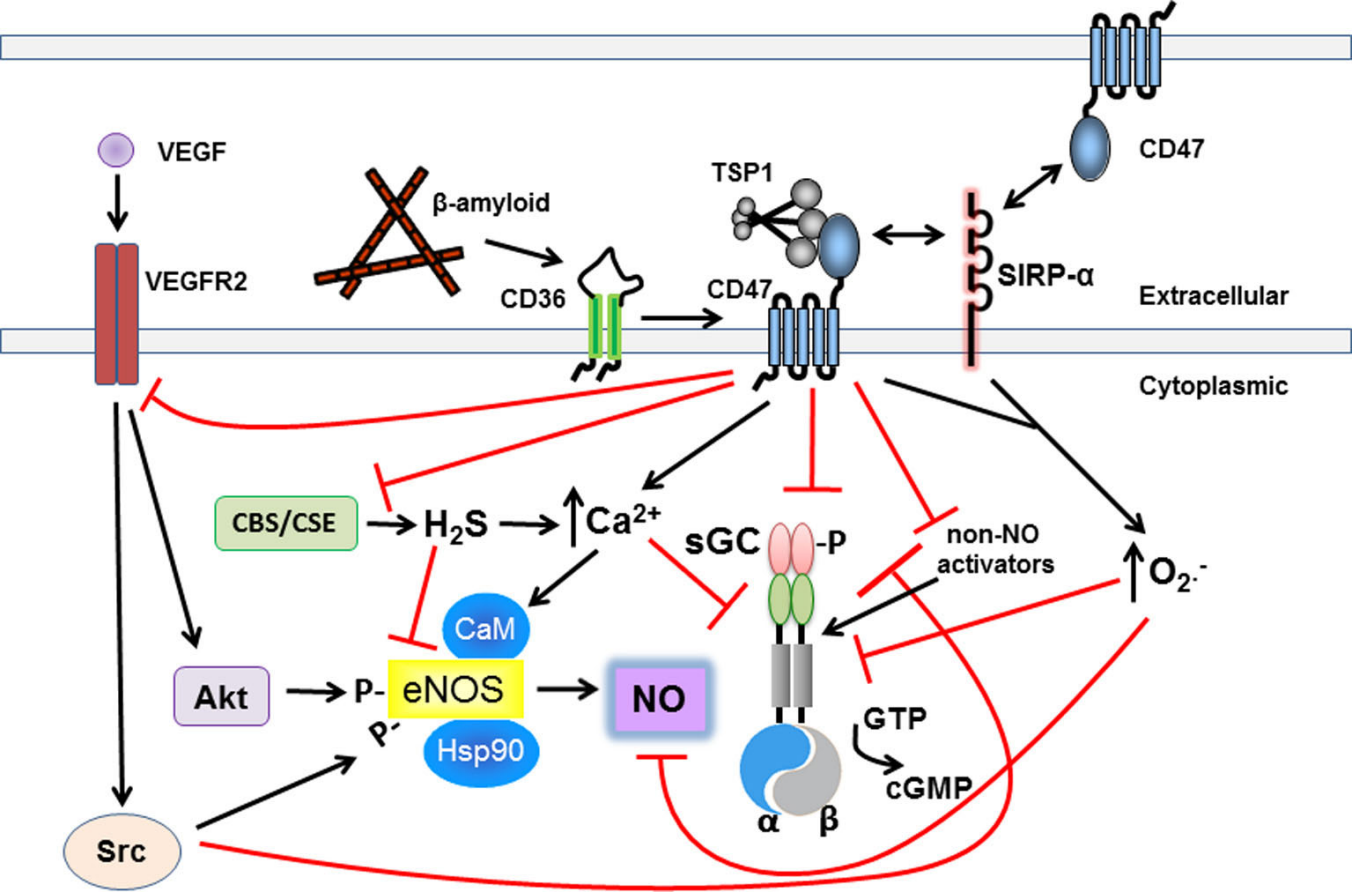
**Matricellular TSP1 via CD47 restricts NO-sGC-cGMP signaling.** TSP1 inhibits the sGC-cGMP axis via multiple mechanisms to circumvent its beneficial effects. Upstream of sGC-cGMP, TSP1 engages CD47 altering cell calcium flux and VEGF receptor signaling to inhibit eNOS-stimulated NO production. TSP1 via CD36 also inhibits eNOS stimulation in endothelial cells by limiting essential free fatty acid uptake (not pictured). TSP1 through CD47, and SIRP-α, stimulates pathologic superoxide production which catabolizes NO. Increased ROS can be expected to alter adversely key protein residues further inhibiting the sGC-cGMP pathway. At the levels of sGC, TSP1 via CD47 directly inhibits sGC and inhibits its sensitivity to NO and non-NO activation. TSP1 can also stimulate increased calcium flux in certain cells which inhibits sGC activation in T cells. TSP1 via CD47 also inhibits synthesis of H_2_S which in turn regulates sGC. Extracellular β-amyloid binds to CD36 and cross talk with CD47 inhibits NO and non-NO activation of sGC. Finally, in vascular smooth muscle cells and platelets TSP1 via CD47 inhibits activation of cGMP-dependent protein kinase.

Several small molecules, including heme-oxidizing agents and calcium have been shown to inhibit sGC activity (Figure 1). The nucleotides ATP, UTP, and CTP have been shown to be competitive, allosteric, or mixed-type inhibitors of sGC (Brandwein et al., 1982; Gille et al., 2004; Ruiz-Stewart et al., 2004; Chang et al., 2005). Heme-oxidizing molecules such as ODQ (1H-[1,2,4]oxadiazolo[4,3-a]quinoxalin-1-one), NS2028 (8-bromo-4H-2, 5-dioxa-3, 9b-diaza-cyclopenta[a]naphthalen-1-one), LY8358369, and methylene blue (Gruetter et al., 1981) inhibit sGC activation by oxidizing its heme moiety (Garthwaite et al., 1995). Heme oxidation is detrimental to enzyme activity for two reasons: (1) Ferric heme has a lower affinity to NO than ferrous heme (Schrammel et al., 1996; Zhao et al., 2000; Fritz et al., 2011), thus NO is unable to activate oxidized sGC as effectively, and (2) Oxidized ferric heme readily dissociates from the enzyme, rendering it insensitive to NO (Fritz et al., 2011). Additionally, heme-free sGC is prone to degradation (Stasch et al., 2006), exacerbating the effect of heme oxidation *in vivo*. The anti-malaria drug artemisinin inhibits NO-stimulated sGC activity with no effect on basal activity (Piatakova and Severina, 2012), although the mechanism of inhibition is unknown. Finally, calcium directly inhibits basal and activated sGC *in vivo* and *in vitro* (Parkinson et al., 1999; Serfass et al., 2001; Kazerounian et al., 2002; James et al., 2009).

## sGC phosphorylation and dephosphorylation

Basal and stimulated sGC activity can both be regulated by post-translational modifications including phosphorylation and dephosphorylation. Depending on the identity of the kinase/phosphatase, sGC can be either inhibited or stimulated via the addition/removal of a phosphate group to/from a Ser, Thr, or Tyr residue. Phosphorylation (Murthy, 2004; Zhou et al., 2008) and dephosphorylation (Ferrero et al., 2000) of the cGMP target PKG, and phosphorylation of the protein tyrosine kinase c-SRC (Meurer et al., 2005; Murthy, 2008) have been shown to be inhibitory (Figure 1). Phosphorylation by protein kinase C (PKC) (Louis et al., 1993) and protein kinase A (PKA) (Zwiller et al., 1981) have been reported to have sGC activating effects. Phosphorylation by Ca^2+/^calmodulin-dependent protein kinase (CaMK) further mediates membrane localization of sGC in cardiomyocytes (Agullo et al., 2005), which may influence the activity state of the enzyme as well.

## sGC thiol oxidation

Human sGC contains 37 Cys residues: 14 in the β and 23 in the α subunit. Consequently, S-nitrosation has been postulated to affect both activation (Fernhoff et al., 2009) and desensitization (Sayed et al., 2008). It was hypothesized that full activation of sGC may only be achieved when a second NO molecule (in addition to the first heme-bound NO) S-nitrosates an unidentified Cys residue on the protein surface (Fernhoff et al., 2009). Biochemical, crystallographic, and mutagenesis studies reveal that S-nitrosation causes NO desensitization of the ferric form of sGC (Sayed et al., 2007). sGC can also be inhibited via mixed disulfide formation of naturally occurring compounds such as cystamine and oxidized coenzyme A (Brandwein et al., 1981). Depletion of the cellular reducing equivalents NADPH and glutathione may cause further sGC thiol oxidation and decreases in enzyme activity (Wu et al., 1992; Mingone et al., 2006). Additional evidence reveals that sGC and protein disulfide isomerase (PDI) co-localize in smooth muscle cells (Heckler et al., 2013). *In vitro* data from COS-7 cell lysates suggests that a disulfide bond exchange between PDI and sGC decreases NO-stimulated activity. The exact mechanism and identification of putative Cys residues involved in this process is the subject of current investigation. Combined, these observations corroborate the importance of the redox state of Cys residues in modulating sGC enzyme activity.

## Oxidative stress in cardiovascular diseases downregulates sGC and eNOS expression

In contrast to the pharmacologic effects of chemical oxidizing agents such as ODQ, the oxidative cellular environment found in many chronic cardiovascular diseases (Cai and Harrison, 2000) also promotes loss of heme in sGC with a subsequent decrease in sGC activity (Wu et al., 1992). These naturally occurring forms of oxidized sGC not only have a lower affinity to NO but are also prone to proteolysis after losing the non-covalently-bound, oxidized heme (Stasch et al., 2006; Fritz et al., 2011). Exacerbating the effect in cardiovascular disease, sGC and eNOS protein expression is down-regulated in ageing cells while the concentration of the reactive oxygen species (ROS) superoxide anion increases, scavenging available NO (Bauersachs et al., 1998). NO and ROS not only regulate sGC activity at the protein level but also influence sGC gene expression at the transcriptional and translational level (Gerassimou et al., 2007). This is relevant in light of new work (discussed below) that describes regulation of NO and ROS by matricellular proteins.

## sGC localization modulates enzymatic activity

Localization of sGC from the cytosol to the membrane may influence enzyme activity through changes in the redox micro-environment and bioavailability of NO. sGC localizes to the plasma membrane in heart tissue in a calcium-dependent fashion (Zabel et al., 2002). Enzyme that fails to localize to the caveolae is more prone to oxidation and consequent heme loss (Linder et al., 2005; Tsai et al., 2012b). Additionally, sGC is more efficiently activated when associated with the membrane because of higher NO availability at that locale. In the cytosol of cardiac myocytes, NO is readily scavenged by myoglobin (Wykes and Garthwaite, 2004). Hence, cellular micro-domains are crucial for sGC function. The protein AGAP1 (ArfGAP protein with a GTPase-like domain, Ankyrin repeats, and a pleckstrin homology domain) may regulate the intracellular distribution of sGC and thus the local delivery of cGMP in mammalian cells (Meurer et al., 2004). The G-protein regulator LGN [Leu-Gly-Asn repeat-enriched protein; also called GPSM2 (G-protein signaling modulator-2) and its homolog AGS3 (activator of G-protein signaling 3)-like] both inhibit sGC activity in cell lysates likely by regulating its cellular localization. Hsp70 and sGC interact in COS-7 cells and co-localization to the plasma membrane has been shown (Balashova et al., 2005). In the central nervous system, the post-synaptic density protein 95 (PSD-95) interacts with the α2β1 isoform of sGC (Russwurm et al., 2001) and targets sGC to the postsynaptic membrane (Brenman et al., 1996). Additionally, in studies employing co-immunoprecipitation, the α1β1sGC enzyme forms a trimeric complex with heat-shock protein 90 (hsp90) and eNOS that localizes to the plasma membrane in aortic smooth muscle cells (Venema et al., 2003). Further confirmation with additional methodologies will be required to verify these results. Recently hsp90 was shown to directly interact with heme-depleted sGC and aid heme incorporation using its inherent ATPase chaperone activity (Ghosh and Stuehr, 2012). It is important to emphasize that most of sGC's interactions with other proteins have only been shown *in vitro* or in cell culture using immunoprecipitation and yeast-to-hybrid assays, and lack confirmation *in vivo*. Further research must provide a physiological context for these observed interactions.

## Pharmacological activation of sGC in cardiovascular diseases: NO donors, stimulators, and activators

Therapeutic agents that target sGC can be divided into three categories: NO donors, sGC stimulators, and sGC activators. Stimulators of sGC synergize and sensitize sGC for activation with NO; activators on the other hand are NO- and heme- independent. NO donor treatment is associated with side effects that include non-specific interactions of NO with other cellular proteins and tolerance development. Thus, there is a need for activators as well as stimulators to directly target sGC and increase cellular cGMP levels in disease states. A prominent sGC stimulator is BAY63-2521 (Riociguat). Riociguat raises the potency of sGC activation by NO and has been approved for the treatment of pulmonary arterial hypertension (Ghofrani et al., 2013). The mode of binding of Riociguat, and other stimulators, to sGC is unknown (Schermuly et al., 2011). In contrast, BAY58-2667 (Cinaciguat)—an sGC activator—works in a NO- and heme-independent manner (Chester et al., 2011; Erdmann et al., 2013) by replacing the heme moiety (Martin et al., 2010). The development of hypotension in patients with acute decompensated heart failure of the Phase IIb clinical trial likely rendered this compound unsuitable for widespread application in this condition (Erdmann et al., 2013). Other heme-replacing activators are BAY 60-2770 and HMR1766 (Ataciguat) (Mendes-Silverio et al., 2012; Kumar et al., 2013). While the mode of binding of sGC activators has been structurally characterized, the binding sites of sGC stimulators remain unknown. Recent evidence, however, suggests that the experimental stimulator YC-1 binds to the βHNOX and/or βHNOXA domains (Purohit et al., 2014).

## Matricellular proteins and emerging sGC regulatory mechanisms

Matricellular proteins are so named because they bind to both structural proteins, such as collagen within the extracellular matrix, and to cells via cell membrane receptors (Lawler and Lawler, 2012). In this capacity they modulate both cellular and matrix processes (Bornstein, 2009). Matricellular proteins themselves do not have known structural function, but they can alter the production and maintenance of matrix directly and through effects upon cells within the matrix (Mosher and Adams, 2012). The role of matricellular proteins in regulating cardiovascular events has been primarily defined in terms of angiogenesis—the process of new blood vessel formation. Angiogenesis (Isenberg et al., 2005a) is required for the healing response that is initiated by wounding and requires days to weeks complete (Miller et al., 2009). Consequently, the kinetics of matricellular signaling, as it relates to the vasculature and blood flow, have been conceptualized as subacute and chronic. Matricellular proteins have a new-found role in regulating acute cardiovascular events including vessel contractility and blood flow. These insights have stemmed from the recently discovered ability of a prototypical member of this class of proteins to rapidly and redundantly limit the canonical NO signaling pathway *in vitro* and *in vivo* (Isenberg et al., 2009b) (Figure 1).

## TSP1 is a prototypical matricellular protein

One of the first identified matricellular proteins that has served as a prototypical example of this class of molecules is thrombospondin-1 (TSP1) (Baenziger et al., 1971; Lawler et al., 1978). TSP1 is the most widely studied member of the thrombospondin family (Bornstein et al., 1991). Five thrombospondins are found in vertebrates (Adams and Lawler, 2011), and of these TSP1 and thrombospondin-2 (TSP2) function as regulatory matricellular proteins in the vascular system. TSP1 is known to interact with at least nine separate cell receptors. TSP1 contains a linear arrangement of functional domains and assembles into a trimeric complex via disulfide bonds near the N-terminus. The N-terminal domain, the central type 1 repeats and C-terminal domain of TSP1 interact with distinct receptors on vascular cells to modulate cell viability (Guo et al., 1997), growth (Taraboletti et al., 1990; Bagavandoss et al., 1993), motility (Vogel et al., 1993; Calzada et al., 2003), contractility (Isenberg et al., 2006b), and gene expression (Kaur et al., 2013). TSP1, directly and indirectly, promotes platelet activation and thrombosis (Bonnefoy et al., 2006; Isenberg et al., 2008e). Given that differential expression of the several TSP1 receptors can change the outcome of its interactions with vascular cells, it is not surprising that contradictory activities have been ascribed to this protein. Early reports linked TSP1 to decreased tumor growth through inhibitory activity on endothelial cell migration and proliferation, with subsequent decreased angiogenesis (Good et al., 1990). Conversely, in arterial smooth muscle cells (Majack et al., 1986) and in retinal pericytes [a smooth muscle like cell associated with capillaries (Scheef et al., 2009)] TSP1 has the opposite effect and promotes proliferation and cell hypertrophy (Wang and Frazier, 1998). Recently, clarification of several contradictory activities ascribed to TSP1 has come from the finding of an intersection between NO signaling and this matricellular protein. These findings have also provided increased understanding of sub-acute and chronic signaling events attributed to TSP1.

## Regulation of sGC and cGMP by thrombospondins

TSP1 limits tumor growth and tumor-associated vascularization (Isenberg et al., 2009b). This well-characterized anti-angiogenic activity was localized to the type 1 repeat domain of TSP1 (Tolsma et al., 1993; Iruela-Arispe et al., 1999), which mediates interaction with the cell surface receptor CD36 (Dawson et al., 1997). TSP1 inhibited growth factor-stimulated corneal angiogenesis in wild type (CD36^+/+^) mice but not in CD36^−/−^ mice (Jimenez et al., 2001). Conversely, in wild type mice treated with a CD36 antibody, TSP1 binding and activation of the receptor was blocked, which increased inflammatory-stimulated corneal angiogenesis (Mwaikambo et al., 2006). More recently, exogenous TSP1, the recombinant type 1 repeat protein domain of TSP1, and a CD36-targeting peptide (based on the proposed CD36 binding site of TSP1) all limited NO-stimulated effects including proliferation and migration of vascular endothelial cells (Isenberg et al., 2005b). TSP1 also rapidly limited NO-mediated activation of sGC to suppress cGMP production. Exogenous TSP1 inhibited sGC sensitivity to NO within 10 min of treatment. Of note, TSP1-mediated inhibition of sGC and downstream suppression of cGMP production is not secondary to alterations in PDE activity. These data indicate that TSP1-CD36 signaling is sufficient to limit NO-sGC signaling in endothelial cells. Arterial smooth muscle cells and platelets also express CD36 in the cell membrane. Treating cells for 10 min with exogenous TSP1 inhibited NO signaling via CD36 as well (Isenberg et al., 2006b, 2008e). The data further suggest that (1) this inhibitory effect occurs in a short period of time, and (2) this is an inhibitory pathway common to all vascular cells. Dose response experiments indicate that primary vascular cells are extremely sensitive to TSP1 with 2.2–22 pmol/L concentrations of TSP1 completely inhibiting NO activation of sGC. Comparison with published dosages in growth factor studies suggests TSP1 inhibition of NO may be a more sensitive signal and may dominate in a given micro-environment *in vivo*.

In a wound healing assay, skeletal muscle angiogenesis stimulated by exogenous NO was potently inhibited by the addition of exogenous TSP1 (Isenberg et al., 2005b). Surprisingly, CD36^−/−^ muscle explants and endothelial cells remained sensitive to TSP1-mediated inhibition of sGC-cGMP signaling (Isenberg et al., 2006a). The finding that CD36^−/−^ cells were susceptible to TSP1-mediated inhibition of NO effects suggested that an alternative cell receptor for TSP1 may play a more important role in this process. TSP1 was demonstrated to functionally interact with the widely expressed cell receptor CD47 (McDonald et al., 2003). CD47 was originally ascribed roles in mediating cell adhesion, platelet activation, macrophage recognition of self (Oldenborg et al., 2000) and integrin signaling (Brown and Frazier, 2001). Our team confirmed that TSP1 binds CD47 with very high affinity (Isenberg et al., 2009a). In contrast to results in CD36^−/−^ tissues, NO-stimulated skeletal muscle angiogenesis in CD47^−/−^ explants is resistant to TSP1 inhibition (Isenberg et al., 2006a). At the level of sGC, CD47^−/−^ endothelial cells have higher basal and NO-stimulated cGMP production than wild type cells. Conversely, TSP1-mediated inhibition of basal sGC and NO-mediated activation is absent in CD47^−/−^ endothelial and vascular smooth muscle cells. Importantly, TSP1 inhibition of sGC activation via CD36 requires the presence of CD47 (Isenberg et al., 2006a). TSP1 also inhibits uptake of free fatty acids via CD36, and this, in turn, limits myristylation of Src-family kinases and downstream activation of eNOS (Isenberg et al., 2007c). Thus, concentrations of TSP1 or TSP1 mimetic drugs such as ABT-510 sufficient to inhibit the fatty acid translocase activity of CD36 can inhibit sGC activation by limiting eNOS activation and subsequent production of the physiological sGC ligand NO (Isenberg et al., 2008f).

At higher concentrations the thrombospondin family members TSP1 and TSP4 can also engage CD47 to inhibit NO-mediated sGC activation (Isenberg et al., 2009a). However, physiological regulation of sGC activity and tissue perfusion was not perturbed in TSP2^−/−^ cells and null mice subjected to ischemic injury. Nonetheless, in certain pathological situations alternative thrombospondins, acting through CD47, may inhibit the NO-sGC-cGMP pathway. As tissue- and time-specific expression of these proteins has been reported, this finding may be of clinical importance. TSP1, and a TSP1-derived recombinant domain containing the CD47 targeting portion of the protein termed E123CaG1 both inhibit NO-mediated sGC activity by as much as >60%, at nanomolar concentrations (Ramanathan et al., 2011). Studies show that under ischemic challenge soft tissue blood flow is lost in CD36^−/−^ animals, whereas it is preserved in CD47^−/−^ animals (Isenberg et al., 2008c), establishing CD47 as the necessary receptor for TSP1 signaling to regulate sGC.

Calcium has been shown to directly inhibit sGC basal function and limit ligand activation of the enzyme *in vivo* and *in vitro* (Parkinson et al., 1999; James et al., 2009). In this context, sGC activation by both stimulators and activators is inhibited by TSP1 (Isenberg et al., 2008a; Miller et al., 2010a) (Figure 1). Human endothelial cells treated with TSP1 (2.2 nmol/L) followed by the calcium ionophore ionomycin demonstrated a decrease in the expected sustained Ca^2+^ release phase. In freshly harvested arteries, *en face* analysis found that TSP1 inhibited ionophore-mediated calcium wave propagation on the endothelium monolayer (Bauer et al., 2010). This is in contrast to TSP1 activity in Jurkat T cells, where both the whole protein and a recombinant C-terminal domain(E123CaG1) that we previously demonstrated interacts selectively with CD47 (Isenberg et al., 2009a), increased cytosolic Ca^2+^ and inhibited sGC (Ramanathan et al., 2011). Upon the interaction of TSP1 or E123CaG1 with the cell membrane, intracellular Ca^2+^ concentrations increased to 300 nM, which in turn activated Ca^2+/^ calmodulin-dependent kinase(s) that putatively inactivated/inhibited sGC through phosphorylation at a yet to be determined residue (Agullo et al., 2005). The inhibitory modification(s) of sGC induced by TSP1-CD47 signaling is stable to disruption of the cell membrane following treatment with E123CaG1 (Ramanathan et al., 2011). TSP1 treatment also increases cytosolic Ca^2+^ in several non-vascular cell types, specifically mast cells and fibroblasts, though the effect this has on NO-sGC-cGMP signaling in these cells is not known (Tsao and Mousa, 1995; Sick et al., 2009). This recent work provides mechanistic insight for a CD47 antibody inhibiting fibronectin, but not histamine-induced Ca^2+^ entry in endothelial cells (Schwartz et al., 1993). Together these data suggest that activated CD47 regulates a non-classical calcium channel in vascular cells.

To date, cell culture experiments in wild type and CD47^−/−^ endothelial and vascular smooth muscle cells and platelets have provided genetic confirmation that TSP1, via CD47, inhibits both basal and NO-stimulated sGC activation (Isenberg et al., 2006a). This process extends to a wide range of mammalian cells as confirmed using vascular cells from mice (Isenberg et al., 2006a), rats (Maxhimer et al., 2009; Yao et al., 2011), pigs (Isenberg et al., 2008d), cows (Bauer et al., 2010; Kaur et al., 2010) and human vascular cells (Isenberg et al., 2005b).

## TSP1 inhibits pharmacologic activation of sGC

Unwanted side effects to NO donor drugs (Rindone and Sloane, 1992) and tolerance following chronic drug exposure (Munzel et al., 2005) have led to efforts to develop sGC stimulators and activators that enhance NO sensitivity and do not require NO at all for enzyme activation. Several of these agents are currently in clinical trials for treatment of cardiovascular diseases (Evgenov et al., 2006; Stasch et al., 2011; Cannon and Pepke-Zaba, 2014). Not surprisingly, TSP1-CD47 inhibition of NO-mediated activation of sGC raised the question of whether TSP1 also inhibited sGC in the presence of sensitizers and activators. In platelets, which also express CD47, a low concentration of TSP1 (2.2 nmol/L) inhibited the disaggregation stimulated by several small molecule activators and stimulators of sGC including YC-1, BAY 41-2272 and meso-porphyrin IX (Miller et al., 2010a). The same concentration of TSP1 inhibited vascular smooth muscle cell relaxation stimulated by these compounds. TSP1 limited the expected increase in cGMP induced by YC1, BAY 41-2272 and PPIX, confirming that sGC was the direct target of this effect. As these agents synergize with both NO and CO to increase sGC activity, these data suggest TSP1 may limit biogas signaling from several sources (see below). Interestingly, the TSP1-derived CD47 activating domain E123CaG1 also inhibited YC-1- and BAY 41-2272-mediated stimulation of sGC. This inhibition could only be reversed by adding back exogenous NO (Ramanathan et al., 2011). This finding may be of clinical relevance, as NO-independent stimulators and activators of sGC are being developed for use in diseases, including pulmonary hypertension (Mittendorf et al., 2009), in which NO signaling is reduced or absent (Rabinovitch, 2007; Stasch and Evgenov, 2013) and, as we have shown, TSP1-CD47 signaling concurrently upregulated (Bauer et al., 2012).

## Alternative ligand activation of the CD36-CD47 complex by β-amyloid inhibits NO- and chemical-mediated sGC stimulation

The secreted protein β-amyloid has been hypothesized to interact with CD36 and CD47 (Bamberger et al., 2003; Wilkinson et al., 2006) (Figure 1). This finding suggested that β-amyloid may act via CD36 and CD47 to inhibit vascular cell sGC-cGMP signaling. Interestingly, β-amyloid protein, and peptides derived from the same, limited essential fatty acid uptake via CD36 (a classic fatty acid translocase). β-amyloid peptides inhibited sGC activation by a primary NO donor and stimulation by BAY 41–2272 in several cell types including bovine endothelial cells, porcine vascular smooth muscle cells and human T cells (Miller et al., 2010b). The inhibitory effect was greatest in human T cells and characterized by an IC_50_ in the nanomolar range. Gene silencing of CD36 attenuated, while gene silencing of CD47 abolished, β-amyloid-mediated inhibition of sGC. Endogenous TSP1 did not further regulate this response. Thus β-amyloid functions as an alternative activator of the CD36-CD47 complex to potently inhibit sGC-cGMP signaling.

## TSP1 inhibits other biogas ligands and activators of sGC

TSP1 signaling through CD47 may also modulate cGMP signaling indirectly via its inhibition of hydrogen sulfide (H_2_S) signaling (Figure 1). H_2_S can regulate NO-sGC-cGMP signaling through regulation of NO synthesis and metabolism (Whiteman and Moore, 2009). TSP1 acting through CD47 in T cells limits the activation-dependent induction of the H_2_S biosynthetic enzymes cystathionine β-synthase (CBS) and cystathionine γ-lyase (CSE) (Miller et al., 2013). TSP1 also inhibits the activity of exogenous H_2_S to activate T cells. Exogenous TSP1 inhibited H_2_S responses in wild type and TSP1^−/−^ T cells but enhanced the same responses in CD47^−/−^ T cells. Engaging CD47 using a CD47-binding peptide derived from TSP1 similarly inhibited signaling. TSP1 signaling via CD47 thereby blocks the autocrine function of H_2_S to increase T cell activation (Miller et al., 2012).

Several mechanisms have been proposed through which H_2_S can regulate NO-sGC-cGMP signaling including regulation of NO synthesis and metabolism (reviewed in Whiteman and Moore, 2009). In pancreatic acini, H_2_S was found to increase Ca^2+^ levels (Moustafa and Habara, 2014). The elevated calcium levels were shown to increase NO synthesis, consistent with the known calcium regulation of eNOS. However as noted above, altered calcium levels can also directly modulate sGC activity. In mice lacking the H_2_S biosynthetic enzyme CSE, the reduced H_2_S levels were associated with increased left ventricular hypertrophy after transverse aortic constriction (Kondo et al., 2013). H_2_S signaling was shown to stimulate VEGF production and Akt-dependent eNOS phosphorylation in this model of pressure overload heart failure, which led to increased cGMP synthesis. Finally, H_2_S induced increased expression of eNOS and iNOS in the vessel walls of treated mice. The anti-thrombotic activity of H_2_S in these mice was partially reversed by administration of the general nitric oxide synthase inhibitor L-N_*G*_-nitroarginine methyl ester (L-NAME) (Kram et al., 2013). A similar induction of eNOS expression by H_2_S was reported in rat corpus cavernosum (Meng et al., 2013).

## TSP1 controls upstream and downstream regulation of the sGC-cGMP axis

Loss of sGC-cGMP signaling in endothelial cells could be caused by direct inhibition of sGC and/or proximal inhibition of NO production at the level of the eNOS. Wild type endothelial cells that express both TSP1 and CD47 display decreased cGMP production compared to CD47^−/−^ cells after stimulation with the eNOS agonist acetylcholine, while treating human endothelial cells with TSP1 inhibited eNOS-mediated production of NO (Bauer et al., 2010). This *in vitro* effect is confirmed in the whole animal as phosphorylation of the key activity-associated eNOS residue serine 1117 is increased in vascular tissues from TSP1^−/−^ mice compared to wild type. In endothelial cells this is mediated by CD47-dependent inhibition of VEGFR2 phosphorylation (Kaur et al., 2010) (Figure 1), which in turn activates Akt to phosphorylate eNOS. TSP1/CD47 signaling also inhibits Src activation, which as mentioned above can inhibit sGC via phosphorylation. Further redundancy in the TSP1-CD47 axis was found in studies of the downstream cGMP-activated kinase (PKG). Phosphorylation of this enzyme can be stimulated in a sGC-independent fashion with the cell permeable analogue 8-bromo-cGMP, and TSP1 inhibits this process (Isenberg et al., 2007a, 2008e). Thus TSP1 redundantly suppresses sGC-cGMP signaling by limiting the upstream production of the sGC ligand NO and by limiting the downstream activation of PKG by cGMP. This later effect is particularly important as it suggests that therapeutic strategies based on NO pro-drugs such as nitrite and NO-independent stimulators of sGC, though relieving inhibition at the level of sGC, cannot overcome upstream and downstream pathway inhibition. Therapeutically these findings imply that the combinatorial approach of NO-independent stimulation of sGC and a CD47 signaling blocker may be the most efficient manner to enhance NO-sGC-cGMP-PKG signaling under the chronic conditions of cardiovascular disease.

## Matricellular protein TSP1 directly stimulates ROS production to further inhibit sGC-cGMP signaling

Cardiovascular disease is characterized by overproduction of pathologic of ROS including superoxide(O^·−^_2_) and hydrogen peroxide (H_2_O_2_) that in turn modify proteins and adversely alter their function (Chen and Keaney, 2012). Conversely, inhibitors of the enzymatic sources of this pathologic ROS are under development for possible therapeutic advantage (Cifuentes and Pagano, 2006). ROS is well known to limit physiologic NO-sGC-cGMP signaling (see above). Hydrogen peroxide induces specific tyrosine phosphorylation of the β1 but not of the α1 subunit of sGC and this may alter enzyme activity (Meurer et al., 2005). ROS can also adversely impact gene function. Treating VSMC with H_2_O_2_ significantly decreased protein levels of the α and β subunits of sGC and inhibited SNP-stimulated cGMP formation (Gerassimou et al., 2007). ROS further limits physiologic NO-sGC-cGMP signaling by reacting with NO (Figure 1). In endothelial cells introduction of intracellular- or extracellular-generated O^·−^_2_ during NO generation resulted in a concomitant increase in oxidative intermediates with a decrease in steady-state NO concentrations (Thomas et al., 2006). Finally, a number of mechanisms exist that serve to buffer and degrade pathologic ROS. Loss of these ROS suppressing enzymatic systems is noted in cardiovascular disease and is associated with increased sGC S-nitrosylation and decreased activity (Choi et al., 2011).

Recently TSP1 has been linked to cardiovascular disease in people. Elevated plasma TSP1 levels are correlated with vasoocclusive crises (Novelli et al., 2012) and peripheral vascular disease (Smadja et al., 2011); tissue TSP1 protein and mRNA levels are overexpressed in the leg muscles of patients with ischemic peripheral vascular disease (Favier et al., 2005), and single nucleotide polymorphisms of TSP1 and TSP4 are associated with higher rates of early myocardial infarction (Narizhneva et al., 2004; Stenina et al., 2005). Given the known roles ROS plays in cardiovascular disease, this suggested that TSP1 may directly participate in ROS production. Hypoxic challenge of human pulmonary arterial endothelial cells increases TSP1 and O^·−^_2_ production while treatment with a CD47 antagonist antibody suppresses this effect (Bauer et al., 2012). Ligation of B-cell CD47 induces mitochondrial dysfunction and ROS production, likely as part of a cell death process (Mateo et al., 2002). Treatment of vascular smooth muscle cells with a low concentration of TSP1 (2.2 nmol/L) increases O^·−^_2_ production within 1 h (Csanyi et al., 2012). In endothelial-denuded arterial rings TSP1 treatment increased CM radical formation as measured with electron paramagnetic resonance. Studies employing pharmacologic inhibitors of enzymatic ROS, siRNA knock down and endothelial free arterial rings from the respective null mice all indicate the source of this O^·−^_2_ is likely the NADPH oxidase 1 (Nox1). TSP1 is a more potent stimulator of ROS compared to the classic activators phorbol myristate acetate and angiotensin II. The CD47 targeting peptide 7N3 that contains the Val-Val-Met sequence also increased VSMC O^·−^_2_ production (Csanyi et al., 2012). Conversely, treating VSMC with a morpholino oligonucleotide to suppress CD47 protein expression (Isenberg et al., 2008d) or an antagonist antibody to block TSP1 binding to CD47, ablated TSP1-mediated O^·−^_2_ production (Csanyi et al., 2012). Thus TSP-CD47 stimulates Nox-derived O^·−^_2_ production in vascular smooth muscle cells.

TSP1 is currently the only identified *soluble* ligand of CD47. However, CD47 can be ligated by the cell membrane protein SIRP-α (Vernon-Wilson et al., 2000). In VSMC, SIRPα has been linked to insulin like growth factor-stimulated ROS production (Xi et al., 2013). New studies in VSMC found that TSP1-mediated O^·−^_2_ production was abrogated in cells treated with a SIRPα morpholino oligonucleotide or a SIRP-α blocking antibody (Yao et al., 2014). Likewise, in renal tubular epithelial cells (rTEC), that we have shown expresses CD47 (Rogers et al., 2012), TSP1 (2.2 nmol/L) treatment increased O^·−^_2_ production which was blocked by treatment with a SIRPα oligonucleotide morpholino and a SIRP-α antagonist antibody (Yao et al., 2014). Together these data show that soluble TSP1 can potently stimulate pathologic O^·−^_2_ production in vascular and epithelial cells and that this was mediated by interactions with both receptors CD47 and SIRP-α. It is likely that the TSP1-SIRPα interaction does not extend to other secreted proteins such as β-amyloid as the fibrillar form of this protein did not inhibit CD47 binding to a SIRP-α-Fc fusion protein (Miller et al., 2010b).

## TSP1-CD47 regulation of sGC-cGMP signaling has effects on blood flow

An immediate effect of sGC activation in arterial smooth muscle cells is uncoupling of actin-myosin interaction and relaxation of the cellular contractile response. This process is central to promotion of blood flow through the vasculature. TSP1 treatment blocks NO- and sodium nitroprusside (SNP)-mediated activation of sGC and subsequent dephosphorylation of myosin light chain 2 (the kinase responsible for vasoconstriction) while increasing immuno-reactive F-actin in cultured VSMC (Isenberg et al., 2007a). *In vitro* studies of VSMC contraction in collagen matrix demonstrated that TSP1 inhibits both NO- and SNP-stimulated relaxation (Isenberg et al., 2007a). In arterial ring myography bioassays, TSP1 (at 0.22 nmol/L) and the CD47-targeting domain E123CaG1 (at 2.2 nmol/L) inhibit endothelial-dependent vasodilation (Bauer et al., 2010). CD47^−/−^ arterial rings are immune to TSP1-mediated inhibition of vasodilation while exogenous TSP1 continues to inhibit Ach-mediated vasodilation in TSP1^−/−^ arterial rings (Bauer et al., 2010). In soft tissues and visceral organs TSP1 inhibits blood flow following ischemia reperfusion injury (IRI) (Isenberg et al., 2008b; Csanyi et al., 2012) while TSP1^−/−^ (Isenberg et al., 2007a, 2008c) and CD47^−/−^ animals (Rogers et al., 2012) display enhanced blood flow following ischemia and IRI. Both eNOS and sGC subunit protein expression levels were comparable in vessels from wild type, TSP1^−/−^ and CD47^−/−^ mice (Bauer et al., 2010) indicating these functional responses are secondary to the acute inhibitory activity of TSP1, via CD47, on the canonical NO-sGC-cGMP pathway.

Based on the above and other work these functional responses may also be, in part, secondary to alternative mechanisms. In VSMC, TSP1 inhibits cAMP-mediated suppression of F-actin organization and inhibits forskolin-stimulated vasodilation of endothelial-free arterial rings (Yao et al., 2011). Alternatively, results in cell cultures suggest that TSP1, via CD47 and/or SIRP-α, could control blood vessel dilation and blood flow through stimulating pathologic ROS. In endothelial-free arterial rings gene knock done of Nox1 abrogated TSP1-mediated inhibition of SNP-stimulated vasodilation (Csanyi et al., 2012). Likewise treatment of arterial rings with the ROS scavenger Tempol corrected TSP1-mediated inhibition of vasodilation (Yao et al., 2014). In animals, an intravenous TSP1 bolus impaired restoration of blood flow following short term (<1 h) ischemia and this was ameliorated by gene silencing of Nox1 or treatment with a CD47 antagonist antibody (Csanyi et al., 2012). Finally, in animals a SIRP-α antagonist antibody, that blocked TSP1-mediated O^·−^_2_ production, enhanced blood flow restoration following 20 min of renal ischemia (Yao et al., 2014).

Null vascular cells display enhanced sGC-cGMP signaling. Employing telemetry monitoring of central blood pressure, TSP1^−/−^ and CD47^−/−^ mice were found to have a widened pulse pressure (Isenberg et al., 2009c), while CD47^−/−^ mice were significantly hypotensive at rest. Similarly, TSP1^−/−^ and CD47^−/−^ animals undergo greater flux in mean arterial blood pressure following an exogenous NO challenge compared to wild type (Isenberg et al., 2009c). It is not clear if variation in autonomic nervous system function plays a role in any of these findings as TSP1^−/−^ and CD47^−/−^ mice show greater sensitivity to post-ganglionic blockade with a greater drop in MAP compared to wild type animals (Isenberg et al., 2009c). In the peripheral microcirculation thermal and NO-stimulated flux in cutaneous blood flow is also greater in TSP1^−/−^ and CD47^−/−^ mice (Rogers et al., 2013).

Under the stress of chronic hypoxia (10% F_*i*_O_2_ for 3 weeks) TSP1^−/−^ mice maintained a more normal cardiovascular profile compared to wild type animals with less change in right ventricular systolic pressure, less right ventricular hypertrophy and less pulmonary vascular smooth muscle cell hyperplasia. This observation was further associated with less pulmonary ROS production (Bauer et al., 2012). TSP1^−/−^ and CD47^−/−^ mice show improved blood flow and less tissue injury in the hind limb compared to wild type following femoral artery ligation and this benefit in tissue perfusion persists with ageing (Isenberg et al., 2007b). However, wild type and TSP2^−/−^ mice display comparable amounts of tissue loss in ischemic soft tissue flaps and comparable diminution in hind limb blood flow after femoral artery ligation (Isenberg et al., 2009a). A lack of a tissue survival and blood flow advantage in TSP2^−/−^ mice is consistent with vascular cell culture experiments where the recombinant TSP2 domain inhibited NO-mediated sGC activation modestly (Isenberg et al., 2009a). This is in contrast to results in TSP1-TSP2^−/−^ mice that show enhanced revascularization following femoral artery ligation compared to wild type animals (Kopp et al., 2006; MacLauchlan et al., 2011).

## TSP1-CD47 regulation of sGC-cGMP signaling has therapeutic implications

TSP1 accounts for approximately 50% of the preformed protein within platelet alpha granules. TSP1 antibodies have shown some effect in tissue models of ischemia (Isenberg et al., 2007d, 2008d). However, it is theoretically more efficient to target CD47. In a human cell line a CD47 antagonist antibody blocks TSP1-mediated inhibition of sGC. Treating full thickness skin grafts with a CD47 antagonist antibody increased graft healing rates in an animal model (Isenberg et al., 2008c). Antisense suppression of CD47 increased sGC-cGMP signaling in VSMC and ischemic tissue survival. In ApoE null mice that demonstrate an accelerated vasculopathy, blocking CD47 expression with morpholino oligonucleotide enhanced ischemic soft tissue survival (Isenberg et al., 2007b). In a severe tissue ischemia model, treating animals with a CD47 antagonist antibody in combination with an NO pro-drug provided additive benefits increasing tissue survival by 100% compared to treatment with the NO pro-drug alone (Isenberg et al., 2009d). This later result is clinically relevant as nitrite is being developed for use in humans (see NIH clinical trials NCT01409122 and NCT01715883 among others). Likewise, interrupting matricellular activation of CD47 enhances liver (Isenberg et al., 2008b) and kidney survival (Rogers et al., 2012) following IRI and ablates pulmonary arterial hypertension in rodents (Bauer et al., 2012). In 5th order pulmonary arteries from the lungs of a patient undergoing transplant for end-stage pulmonary arterial hypertension the TSP1-CD47 axis was induced while NO-dependent and independent vasodilation was almost abolished (Rogers et al., 2013; Published online Feb 3, 2014), confirming in human disease activation of the TSP1-CD47 inhibitory axis is concurrent with loss on NO-sGC-cGMP signaling. Taken together basic and clinical studies suggest that a more comprehensive way to treat chronic cardiovascular disease would be to combine sGC activation with disruption of the TSP1-CD47 pathway as a novel therapeutic strategy.

## Funding

This work was supported by NIH grants R01HL-108954 and 1R01HL112914-01A1 and American Heart Association grants 11BGIA7210001 (Jeffrey S. Isenberg) 10SDG2600345 (Elsa D. Garcin), 13PRE17000045 (Franziska Seeger) and 13POST14520003 (Natasha M. Rogers) and by the Intramural Research Program of the NIH/NCI (David D. Roberts). This work was further supported by the Institute for Transfusion Medicine, the Hemophilia Center of Western Pennsylvania and the Vascular Medicine Institute (Jeffrey S. Isenberg).

### Conflict of interest statement

Jeffrey S. Isenberg is Chair of the Scientific Advisory Boards of Vasculox, Inc. (St. Louis, MO) and Radiation Control Technologies, Inc. (RCTI, NY, NY) and holds equity interest in the same. The authors declare that the research was conducted in the absence of any commercial or financial relationships that could be construed as a potential conflict of interest.
